# Supplementary material for: Effect of Ionizing Radiation from Computed Tomography on Differentiation of Human Embryonic Stem Cells into Neural Precursors
Source: Int J Mol Sci. 2019 Aug 10;20(16):3900. doi: 10.3390/ijms20163900 (PMC6720494; doi:10.3390/ijms20163900)
Supplement: Supplementary file 1 [file ijms-20-03900-s001.pdf]

**Supplementary Information**

# Effect of Ionizing Radiation from Computed Tomography on Differentiation of Human Embryonic Stem Cells into Neural Precursors.

Christine Hanu[1]*, Burk Loeliger[1]*, Irina V. Panyutin[1], Roberto Maass-Moreno[1], Paul Wakim[2], William F. Pritchard[1], Ronald D. Neumann[1], and Igor G. Panyutin[1].

**Supplementary Figure Legends**

Figure 1S. 3D torso tissue-simulating phantom that was used for CT scans of hESCs in T-75 cell culture flasks placed in its abdominal cavity.

Figure 2S. Colonies of hESCs stained with OCT4 (green) and 53BP1 (red) antibodies showing pluripotency and repair foci after exposure to radiation.

Figure 3S. Neural precursor cells (Day 25) stained with DAPI (blue) and 53BP1 antibodies (red) showing repair foci after exposure to radiation.

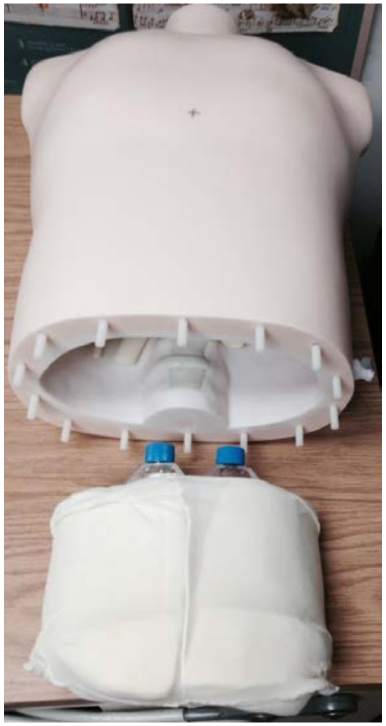

Figure 1S. 3D torso tissue-simulating phantom that was used for CT scans of hESCs in T-75 cell culture flasks placed in its abdominal cavity.

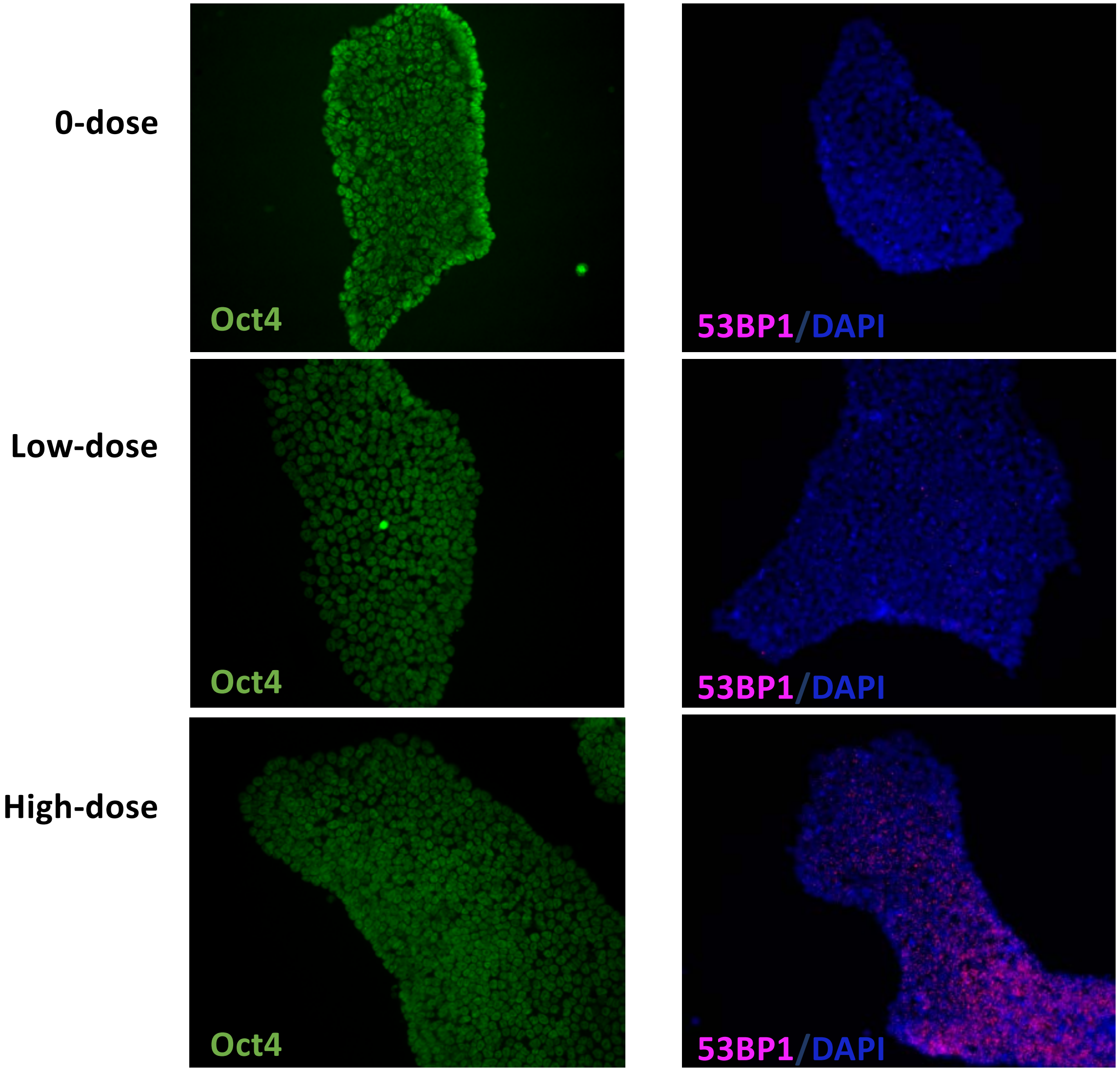


Figure 2S . Colonies of hESCs stained with OCT4 (green) and 53BP1 (red) antibodies showing pluripotency and repair foci after exposure to radiation.

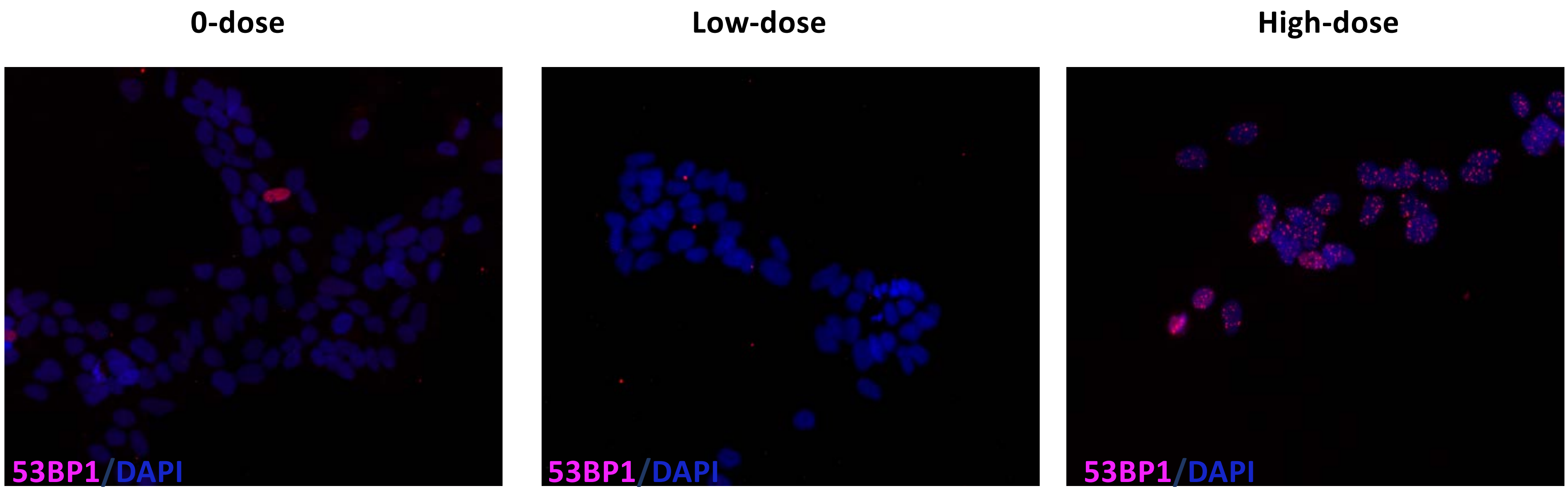


Figure 3S . Neural precursor cells (Day 25) stained with DAPI (blue) and 53BP1 antibodies (red) showing repair foci after exposure to radiation.
